# Case Report: Acute pyelonephritis and hearing loss in scrub typhus

**DOI:** 10.12688/f1000research.18129.2

**Published:** 2019-07-05

**Authors:** Sivaranjini Venketesan, Dheeraj Jain, Stalin Viswanathan, Murugesan Sivagurunathan Gayathri

**Affiliations:** 1 Department of General Medicine, Indira Gandhi Medical College & Research Institute, Pondicherry, Puducherry, 605009, India; 2 Department of General Medicine, Jawaharlal Institute of Postgraduate Medical Education and Research (JIPMER)., Pondicherry, Puducherry, 605009, India; 3 Department of Radiology, Indira Gandhi Medical College & Research Institute, Pondicherry, Puducherry, 605009, India

**Keywords:** scrub typhus, acute pyelonephritis, urinary tract infection, hearing loss

## Abstract

Acute pyelonephritis is a common renal manifestation in patients with diabetes. A 52-year-old diabetic lady presented with loin pain, dysuria, and fever and urinary incontinence that had begun seven and three days prior to presentation respectively. She was treated with escalating spectra of intravenous antibiotics without improvement. Urine and blood cultures were sterile, while radiological investigations were suggestive of pyelonephritis. Mild hepatic dysfunction prompted consideration of scrub typhus and she improved with empirical doxycycline. Scrub IgM was later confirmed to be positive. In conclusion, local prevalence of systemic infections such as rickettsioses should always be considered in diabetics with fever, even if symptoms and signs otherwise suggest typical diabetes-related infections. We, therefore report a case of acute pyelonephritis caused by scrub typhus which has not been previously described in English medical literature.

## Introduction

Among patients with diabetes mellitus, the urinary tract is the most common site of infection ^1^. Urinary tract infections (UTI) are either related to the upper or lower urinary tract. Acute and chronic pyelonephritis are upper UTIs ^1^. Bacteria ( *Escherichia coli*), viruses (Adenovirus), fungi ( *Mucor*), and mycobacteria ( *Mycobacterium tuberculosis*) commonly cause upper UTIs in diabetes ^1^. *Orientia tsutsugamushi* (scrub typhus) has never been reported to cause pyelonephritis in English medical literature.

## Case report

A 52-year-old grandmother of Indian origin, non-compliant to insulin for six months, presented to the Emergency Department of our hospital with fever and rigors, vomiting, headache, bilateral leg pain and myalgia, which had persisted for one week and urinary incontinence for the prior three days. She was unemployed, did not consume alcohol and had had no exposure to rodents or mite bites. Apart from over-the-counter antipyretics, she had neither consulted a health practitioner nor had she received antibiotics. On examination, she was conscious, oriented, toxic, febrile, drowsy, dehydrated with slurred speech, with body-mass index 20.2 kg/m ^2^, tachycardia, orthostatic hypotension, diminished hearing, with right renal angle fullness and tenderness. Initial investigations ( Table 1) revealed random sugars 435mg/dL, normal renal functions, ketonuria and glycosuria without pyuria, sinus tachycardia (electrocardiogram), and normal echocardiography. There were no malarial parasites on the peripheral smear. Arterial blood gas showed respiratory alkalosis with metabolic acidosis. Intravenous ceftriaxone 2g OD, intravenous fluids, insulin, acetaminophen 500mg three times a day, multivitamins (B12 1000µg, thiamine 100mg, pyridoxine 100mg, riboflavin 5mg and folate 5mg), pantoprazole 40mg, and domperidone 10mg were commenced for probable acute pyelonephritis. On day 3, piperacillin/tazobactam 4.5g every 8 hours and fluconazole 300mg once a day (OD) were substituted for ceftriaxone 2 g OD; oral amitriptyline 25mg was added to treat the patient’s painful neuropathy.

**Table 1.** Investigations of the patient during hospital stay and follow-up. Normal ranges for each test are provided. ESR - erythrocyte sedimentation rate.

	Day 1	Day 3	Day 5	Day 7	Follow up
Sodium (136–142 mmol/ L)	125	129			
Total bilirubin) (1.1–2.4mg/dL)			1.87		2.25
Direct bilirubin (0.1–0.4mg/dL)			0.3		0.4
Total protein (6.3–7.9g/dL)			5.2		6.9
Albumin (3.5–5.5g/dL)			2.8		3.8
Aspartate aminotransferase (12–38U/L)			175		72
Alanine aminotransferase (7–40U/L)			64		63
Alkaline phosphatase (35–100U/L)			299		262
Creatinine (0.5–0.9mg/dL)	0.9				
Hemoglobin (11.7–15.7g/dL)	10.8	9.0	8.9	7.2	
Total leukocyte count (4–11 ×10⁹/L)	13.1	8.9	7.0	6.8	
Platelets (150–450 ×10⁹/L)	202	311	362	368	
ESR (0–20 mm/hour)	112	90	40		

**Figure 1.**
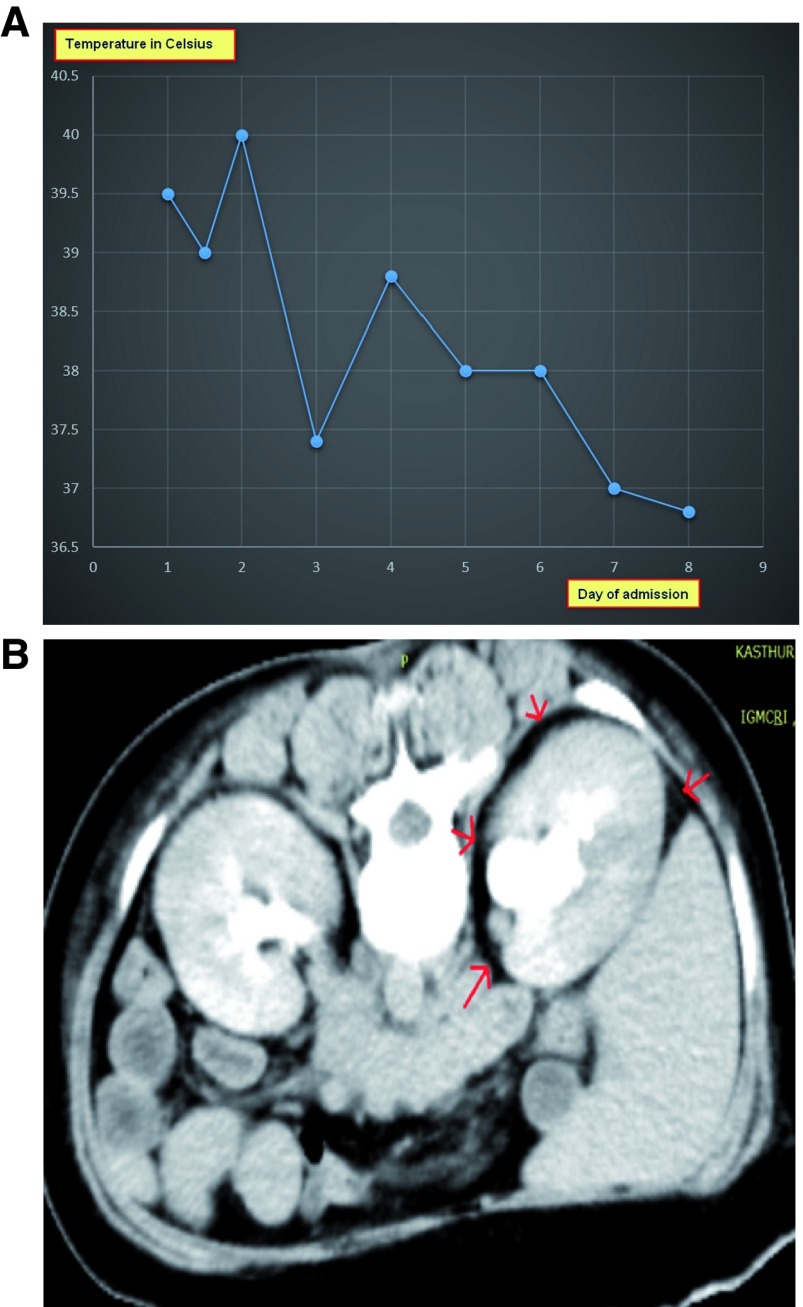
( **A**) Fever spikes plotted from day 1 to day 5. ( **B**) Computed tomography scan revealing bilateral renomegaly and mild fat stranding in the right kidney.

Blood and urine cultures were sterile. Ultrasonogram showed hepatomegaly and bilateral bulky kidneys. She developed diarrhea on day 4. Hence, piperacillin was discontinued after 36hrs even though there appeared to be a partial defervescence; diarrhea subsided after discontinuing piperacillin. Her toxemia and prostration persisted, and she needed assistance to the toilet in view of extreme weakness, but had no focal neurological deficits. On day 5, she was initiated on meropenem 1g every 8 hours and linezolid 600mg every 12 hours for persisting fever ( Figure 1A). Liver function tests showed elevated transaminases; hence probable rickettsioses was suspected and empirical doxycycline 100mg twice a day was initiated on day 6. The next day, Scrub Typhus Detect IgM (InBios International) done at a private laboratory returned positive with an OD of 1.732 (Cut-off: <0.500). Intravenous antibiotics were therefore discontinued and there was no recurrence of fever thereafter.

Abdominal computed tomography (CT) on the 8 ^th^ day showed bilateral bulky kidneys with mild perinephric fat stranding ( Figure 1B), thus confirming the provisional diagnosis. The pancreas was normal. She also had left-sided proliferative diabetic retinopathy and bilateral sensorineural hearing loss (average of 75dB and 90dB in the right and left ear respectively). She then completed a 7-day course of doxycycline 100mg twice a day and was advised another week’s therapy at home. She had had no fever thereafter. On follow up, liver function test (LFT) had improved ( Table 1), as did her hearing (47 dB in the right and 87 dB in the left) which favored our diagnosis of probable acute scrub typhus.

## Discussion

Diabetes mellitus, due to hyperglycemia, ketoacidosis, vascular insufficiency, and impaired neutrophil and monocyte function, makes patients prone to UTIs ^1^. Diagnosis of acute pyelonephritis (APN) clinically is a syndrome of fever, chills, vomiting, and flank pain associated with pyuria, and is often radiologically confirmed ^2^. In a prospective study, only 1/4 ^th^ patients had a positive urine culture and only 65% had pyuria ^2^, echoing the findings in our patient. In total, 14 among 223 patients had diabetes. Even though our patient did not have pyuria, symptoms/signs in a poorly controlled diabetic led us to a diagnosis of APN and empirical treatment was instituted for the same. Renal abnormalities in scrub typhus range from simple proteinuria/hematuria to acute kidney injury and occasionally, chronic kidney disease ^3^. Our patient had glycosuria and positive microalbuminuria (92µg/mg). Mechanisms postulated for renal involvement include rickettsiae-related vasculitis, tubular interstitial proliferation, and tubular necrosis ^3^. APN in scrub typhus has been reported only once, in Chinese medical literature in a 56-year-old Chinese lady who had urgency, flank pain and an eschar ^4^.

Diabetes is a risk factor for scrub typhus-induced acute kidney injury. Since leukocytosis reduced with ceftriaxone without adequate fever response, we presumed poor control of bacterial/fungal infection and treated her with fluconazole and piperacillin/tazobactum. Since LFT could not be performed prior to day 4 due to technical reasons, rickettsioses were not suspected. Even though our locality is a high-prevalence area for scrub typhus, focal renal signs and symptoms led us to think otherwise ^5^. A convalescent titer of Scrub IgM could not be done due to its unavailability and need for out-of-pocket expenses. PCR was not available. We also erred in contributing her hearing impairment to be the result of her toxemia and poor health. Pure tone audiometry was done 48 hours after doxycycline when the patient became self-ambulatory. Improvement of her hearing loss, albeit partial, two weeks after discharge suggests that scrub typhus could have also contributed to her hearing impairment ^6^. Abdominal CT was also done after doxycycline therapy-whether findings are milder than expected is also debatable. Hypoalbuminemia and rapidly falling hemoglobin over seven days without overt blood or volume loss, could be attributed to hemoconcentration following scrub typhus-related capillary leak syndrome that was observed at initial presentation, and reverted to premorbid levels after fluid supplementation and antibiotics. In retrospect, fever, absence of pyuria, sterile urine, capillary leak syndrome, primary respiratory alkalosis, and hearing loss in a patient with high sugars and ketonuria should have made us think of an alternative etiological diagnosis. The pattern of LFT derangement did not suggest a biliary infection and pancreatic imaging was normal. Also, fever did not recur after initiating doxycycline and discontinuing other intravenous antibiotics which would favor a rickettsial infection rather than a systemic bacterial sepsis.

UTIs in diabetes are common, but scrub typhus as a probable cause of UTI/pyelonephritis has hitherto been unreported in English medical literature. Atypical organisms causing pyelonephritis should be considered in patients with multisystem involvement and in those with a UTI but without pyuria. Furthermore, local prevalence of systemic infections such as rickettsioses should always be considered in diabetics with fever, even if symptoms and signs otherwise suggest typical diabetes-related infections.

## Consent

Written informed consent for publication of their clinical details and clinical images were obtained from the patient.

## Data availability

All data underlying the results are available as part of the article and no additional source data are required.
