# Supplementary material for: Case Report: Acute pyelonephritis and hearing loss in scrub typhus
Source: F1000Res. 2019 Jul 5;8:312. Originally published 2019 Mar 20. [Version 2] doi: 10.12688/f1000research.18129.2 (PMC6613477; doi:10.12688/f1000research.18129.2)
Supplement: Peer review history

# Peer review: Reviewer response for version 2

**DOI:** 10.5256/f1000research.21739.r50808

**Published:** 2019-07-05

**Authors:** Tri Wangrangsimakul

**Affiliations:** 1 Mahidol-Oxford Tropical Medicine Research Unit, Faculty of Tropical Medicine, Mahidol University, Bangkok, Thailand; 2 Centre for Tropical Medicine and Global Health, Nuffield Department of Clinical Medicine, University of Oxford, Oxford, UK

The authors have revised the article and provided further useful information that improves the overall quality of the report. The lack of a convalescent sample for serological testing or other confirmatory tests remain a weakness. However, the additional details on risk factors and diagnostic testing should allow readers to draw a conclusion on the likelihood of scrub typhus infection causing pyelonephritis in this patient.

I confirm that I have read this submission and believe that I have an appropriate level of expertise to confirm that it is of an acceptable scientific standard.

# Peer review: Reviewer response for version 1

**DOI:** 10.5256/f1000research.19827.r48908

**Published:** 2019-05-28

**Authors:** Tri Wangrangsimakul

**Affiliations:** 1 Mahidol-Oxford Tropical Medicine Research Unit, Faculty of Tropical Medicine, Mahidol University, Bangkok, Thailand; 2 Centre for Tropical Medicine and Global Health, Nuffield Department of Clinical Medicine, University of Oxford, Oxford, UK

The authors report a case of a 52 year old diabetic lady with complications, presenting with pyelonephritis. Cultures were negative and she was treated with antibiotics that were regularly escalated. She was also found to have bilateral sensorineural deafness on audiometry. Elevated liver enzymes prompted testing for scrub typhus and the IgM came back positive, leading to initiation of doxycycline and defervescence within hours.

 For any case report, the diagnosis must be as robust as possible and an effort made to rule out other possible diagnoses. I agree with the other review that malaria should be ruled out. Other diagnoses to be excluded include leptospirosis, biliary infection or pancreatitis. Other background information would be useful e.g. antibiotics used pre-admission, occupation, freshwater contact, rodent contact, agricultural work, insect bites, alcohol intake and past history of scrub typhus.

 The basis for the diagnosis of scrub typhus used in this report is limited. A "'Scrub IgM positive" does not inform the reader of what serological test or method was used, where the test was performed, what diagnostic cut-off was used to determine a positive result and whether this cut-off has been validated for the population which the patient belongs to. I suspect the test used was the InBios Scrub Typhus IgM ELISA, in which case the OD value and how the diagnostic cut-off was reached should be stated so the reader can form their own opinion on the strength of diagnosis.

 The diagnosis of scrub typhus is not straightforward ^1^. Antibody-detection tests (IFA, ELISAs) have inherent weaknesses, such as the presence of circulating antibodies in individuals within an endemic area where repeated exposure occurs, leading to false positive results. Acute and convalescent samples can give a clearer picture and a rise in antibody titre to >= 4 times is usually part of the diagnostic criteria. Is a convalescent blood sample available to compare IgM serology results? Culture and PCR will be more specific and will greatly improve the certainty of diagnosis but may not be available.

 Scrub typhus can cause AKI, glomerulonephritis and nephrotic syndrome so pyelonephritis is possible ^2^^,^^3^. However, the diagnostic case for scrub typhus must be more robust than currently presented. Additionally, one could argue that the general trend was that she was improving when doxycycline was initiated on day 6 as observed on the fever chart, which would go against severe scrub typhus with a renal complication.

 Two main lessons can be drawn from this report. Firstly, in regions where scrub typhus is endemic and accurate diagnostic assays are unavailable, empirical treatment with doxycycline should be considered. Secondly, antibody-detection assays for scrub typhus have inherent weaknesses and until paired antigen/IgM tests are available, acute and convalescent samples are recommended for diagnostic confirmation. In this report, the evidence for scrub typhus infection is currently limited and the conclusion that this diagnosis is established should be delayed until further diagnostic information is available.

I confirm that I have read this submission and believe that I have an appropriate level of expertise to confirm that it is of an acceptable scientific standard, however I have significant reservations, as outlined above.

## References

[1] : State of the art of diagnosis of rickettsial diseases: the use of blood specimens for diagnosis of scrub typhus, spotted fever group rickettsiosis, and murine typhus. *Curr Opin Infect Dis*.29(5) : 10.1097/QCO.0000000000000298 433-9 10.1097/QCO.0000000000000298 27429138PMC5029442

[2] : Clinical manifestations of scrub typhus. *Transactions of The Royal Society of Tropical Medicine and Hygiene*.2017;111(2) : 10.1093/trstmh/trx017 43-54 10.1093/trstmh/trx017 28449088

[3] : A Comparative Study of the Pathology of Scrub Typhus (Tsutsugamushi Disease) and Other Rickettsial Diseases. *Am J Pathol*.1945;21(4) :603-81 19970829PMC1934171

# Response

**Published:** 2019-06-26

**Authors:** Stalin Viswanathan

**Affiliations:** JIPMER, India

Dear Sir,

Malarial parasites were not visualized on the peripheral smear on at least 2 occasions. Nevertheless, there was no hepatosplenomegaly, discolored urine, jaundice, hyperbilirubinemia and thrombocytopenia to suggest malaria.

The same goes for biliary infection. The lady did not have pain suggestive of pancreatitis and computed tomography of pancreas was normal. We did not perform lipase.

Leptospirosis was considered even without her exposure to rain, water, or rodents but the thought did not persist in the absence of renal failure and thrombocytopenia and lack of response to ceftriaxone in the first three days.

Background information regarding her employment and others have been added to the manuscript.

The diagnostic kit and the cutoff values have been mentioned in the manuscript. Convalescence titers could not be performed due to the cost of performing it in an outside lab.

We would not generally agree that she was improving till day 5 even without doxycycline since the toxemia persisted that made us initiate meropenem and linezolid. Her symptoms did not recur on stopping these drugs and initiating doxycycline.

Culture and PCR were not available. This has been added to the text.

# Peer review: Reviewer response for version 1

**DOI:** 10.5256/f1000research.19827.r45955

**Published:** 2019-04-03

**Authors:** Ravindra Prabhu Attur, Dharshan Rangaswamy

**Affiliations:** 1 Department of Nephrology, Kasturba Medical College, Manipal Academy of Higher Education, Manipal, India

The authors describe a rare reported manifestation of Scrub Typhus namely acute pyelonephritis and hearing loss which improved with treatment. Acute pyelonephritis has been reported once as a case report from China and hearing loss in another six cases. The case is well described and convincing in that clinical features favor acute pyelonephritis. However since authors are describing a manifestation for the first time a more detailed account would have been better. For example, in view of negative cultures, treatment received prior to entry could have been listed. The authors base their conclusion on a chance sending of scrub IgM which came positive whereas learning points which serve as pointers to the diagnosis may be mentioned in discussion. The presence of liver dysfunction, anemia and fever would have merited investigation with other common tropical infections such as malaria, which can also involve the kidneys.

 Unusual features in this description are absence of eschar, thrombocytopenia for scrub typhus and absence of pyuria. The absence of pyuria in 1/3 ^rd^ cases as observed in a previous prospective study, had only 6% of diabetics in their cohort and the diagnosis of acute pyelonephritis was mainly based on clinical criteria ^1^. The sensitivity and specificity for diagnosis of acute pyelonephritis on urine investigations are: urinalysis (>10 WBCs/high power field), 58%–82% and 65%–86%; positive leukocyte esterase, 74%–96% and 94%–98%; nitrite, 35%–85% and 92%–100%; and leukocyte esterase plus nitrite tests, 75%–84% and 82%–89% respectively ^2^^,^^3^^,^^4^^,^^5^. The sensitivity and specificity for diagnosis for acute pyelonephritis based on perinephric fat stranding was 72% and 60%. It is affected by age, sex and renal function and may not be a particularly useful tool for the diagnosis of acute pyelonephritis ^6^. Bilateral enlarged kidneys, renal angle tenderness with associated vomiting are also observed in patients with infection or drug related acute interstitial nephritis. However, the absence of pyuria points against the diagnosis.

 Certainly, scrub typhus can cause inflammation of organs due to endothelial infection, vasculitis and increases endothelial permeability. This report highlights the importance of keeping a high index of suspicion for this easily treatable disease especially in endemic areas. The article can be accepted as approved with caveats stated above.

We confirm that we have read this submission and believe that we have an appropriate level of expertise to confirm that it is of an acceptable scientific standard.

## References

[1] : Acute pyelonephritis in adults: a case series of 223 patients. *Nephrol Dial Transplant*.2012;27(9) : 10.1093/ndt/gfr810 3488-93 10.1093/ndt/gfr810 22344777

[2] : Optimized urinary microscopy for assessment of bacteriuria in primary care. *J Fam Pract*.1990;31(2) :153-9; discussion 159 2199600

[3] : Diagnosis and treatment of uncomplicated urinary tract infection. *Infect Dis Clin North Am*.1997;11(3) :551-81 937892310.1016/s0891-5520(05)70373-1

[4] : Laboratory techniques for detection of urinary tract infection and assessment of value. *Am J Med*.1983;75(1B) :79-84 634934710.1016/0002-9343(83)90076-1

[5] : Evaluation of the leukocyte esterase and nitrite urine dipstick screening tests for detection of bacteriuria in women with suspected uncomplicated urinary tract infections. *J Clin Microbiol*.1999;37(9) :3051-2 1044950510.1128/jcm.37.9.3051-3052.1999PMC85454

[6] : Laboratory evaluation of leukocyte esterase and nitrite tests for the detection of bacteriuria. *J Clin Microbiol*.1985;21(5) :840-2 399811810.1128/jcm.21.5.840-842.1985PMC271794

[7] : Perirenal fat stranding is not a powerful diagnostic tool for acute pyelonephritis. *Int J Gen Med*.2017;10: 10.2147/IJGM.S133685 137-144 10.2147/IJGM.S133685 28507449PMC5428832

# Response

**Published:** 2019-06-26

**Authors:** Stalin Viswanathan

**Affiliations:** JIPMER, India

Dear Sir,

The lady did not receive any prior treatment. The details have been included.

It was not a chance sending of Scrub IgM. That facility is not available at our institution. Since LFT was done only on day 4, we could send a sample for Scrub IgM to a private lab only on day5.

Malarial parasites were not visualized on peripheral smear. Moreover, the LFT dysfunction was without hyperbilirubinemia.

All these details were not mentioned since this manuscript was submitted to another journal previously with a word count of 1000, and such information fell prey to the editing keys.

Unusual was the absence of eschar, but it is well known in Indian populations that the incidence of eschar is varying between 4-46% (Vivekanandan *et al*, 2010).

Thrombocytopenia was expected in a case of scrub typhus, and such a finding would have enabled us to pursue a diagnosis of scrub typhus earlier, even before the LFT.
